# Study of Immobilization Procedure on Silver Nanolayers and Detection of Estrone with Diverged Beam Surface Plasmon Resonance (SPR) Imaging

**DOI:** 10.3390/bios3010157

**Published:** 2013-03-19

**Authors:** Alina Karabchevsky, Lev Tsapovsky, Robert S. Marks, Ibrahim Abdulhalim

**Affiliations:** 1 Electro-Optic Engineering Unit and Ilse Katz Institute for Nanoscale Science and Technology, Ben-Gurion University of the Negev, Beer-Sheva 84105, Israel; E-Mail: abdulhlm@bgu.ac.il; 2 Environmental Engineering Unit, Ben-Gurion University of the Negev, Beer-Sheva 84105, Israel; E-Mail: liovats82@gmail.com; 3 Biotechnology Engineering, National Institute for Biotechnology in the Negev and Ilse Katz Institute for Nanoscale Science and Technology, Ben-Gurion University of the Negev, Beer-Sheva 84105, Israel; E-Mail: rsmarks@bgu.ac.il; 4 School of Materials Science and Engineering, Nanyang Technological University, 637722, Singapore

**Keywords:** optical immunosensor, immobilization, estrone, endocrine disruptor, SPR imaging

## Abstract

An immobilization protocol was developed to attach receptors on smooth silver thin films. Dense and packed 11-mercaptoundecanoic acid (11-MUA) was used to avoid uncontrolled sulfidization and harmful oxidation of silver nanolayers. N,N*'*-dicyclohexylcarbodiimide (DCC) and N-hydroxysuccinimide (NHS) were added to make the silver surfaces reactive. A comparative study was carried out with different immersion times of silver samples in 11-MUA solutions with different concentrations to find the optimum conditions for immobilization. The signals, during each step of the protocol, were analyzed with a refractometer based on the surface plasmon resonance (SPR) effect and luminescence techniques. Molecular interactions at the surfaces between the probe and target at the surface nanolayer shift the SPR signal, thus indicating the presence of the substance. To demonstrate specific biosensing, rabbit anti-estrone polyclonal immunoglobulin G (IgG) antibody was immobilized through a linker on 47 nm silver layer deposited on SF11 glass. At the final stage, the representative endocrine disruptor—estrone—was attached and detected in deionized water with a diverging beam SPR imaging sensor.

## 1. Introduction

Optical biosensors based on surface plasmon resonance (SPR) are a type of-surface sensitive evanescent wave device used for label-free detection. Molecular interactions at the surfaces between the probe and the target shifts the SPR signal, thus indicating the presence of the substance. The high sensitivity and relatively low cost makes the SPR biosensors widely used in the field of low concentration sensing. It has been widely applied in the detection of chemical and biological analytes [1]. SPR sensor instrumentation occupies different methods of optical excitation of surface plasmons [2], including attenuated total reflection (ATR) using prism coupling, grating coupling or waveguide coupling, extraordinary transmission (EOT) through narrow metallic nano-openings, excitation of localized surface plasmons (LSPR) with surface enhanced Raman scattering (SERS) or surface enhanced fluorescence (SEF). SPR sensors were applied to detect a large variety of biological and biochemical entities for medical and environmental applications [3].

The development of biosensing devices requires methods for immobilization of specific receptors. In addition to the specificity, immobilization is important to maintain constant environmental conditions of the sensor. In the case of silver, it has to be protected against the uncontrolled sulfidization, changes in pH and harmful oxidization. Thiol molecules are widely used with gold immobilization, as summarized in Table 1. 

**Table 1:** List of Mercaptoundecanoic acid (MUA) solutions used with gold surfaces.

Reference	Concentration [mM]	Incubation [h]	Solvent
[4]	10	24	Ethanol
[5]	10	20–24	Ethanol
[6]	5	2	Ethanol
[7]	2	12	Ethanol
[8]	1	18	Ethanol
[9,10,11]	1	24	Ethanol
[12,13]	150	12	Glycerol/Ethanol
[14]	10	24	Ethanol
[15]	1	12	Ethanol
[16]	20	16	Ethanol
[17]	5	2	Ethanol
[18]	10	12	Ethanol
[19]	10	24	Ethanol
[20]	1	4	Ethanol

old is usually used in SPR biosensors, since it is an inert metal and enables easy immobilization using a self-assembly monolayer (SAM)—spontaneous binding of thiols to metal—which is widely used to conjugate biomolecules to the metal layers. However, gold experiences a poor attachment to the glass substrates and needs a few nanometers of Ti or Cr deposited on glass prior to the deposition of a gold layer, which broadens the SPR signal. SPR signals achieved from the silver have a very sharp curve and show better sensitivity. Since silver suffers form uncontrolled sulfidization and harmful oxidation, it has to be protected in addition to the immobilization procedures. Care must be taken in choosing the material that will not deteriorate the sensitivity of the sensor. The common protective layers are a variety of oxides (SiO_2_, ZrO_2_) and polymethyl methacrylate (PMMA). PMMA, which is a transparent thermoplastic, was reported in studies on extraordinary transmission (EOT) through the narrow nano slits in silver [21,22,23] as a protection layer. It was found that up to the 15 nm of PMMA, the sensitivity of the EOT sensor is not affected [23]. A 21 nm layer of SiO_2_ was deposited on smooth silver thin films for the SPR, based on attenuated total reflection (ATR) using a prism coupling sensor and the diverged beam SPR imaging (DBSPRI) approach [24]. These layers make the immobilization more difficult, and therefore, gold is a popular metal used in SPR biosensors, although it is less sensitive and its signal is wider than those of silver, especially in the visible range. Recently, enhancement of fluorescent signals by a factor of about 32 was demonstrated with immobilized fluorescent receptor anti-rabbit IgG (1/100 antibody/PBS) on silver columnar thin films using thiol [25]. 

We report here for the first time on an optimized procedure for immobilization of receptors on silver nano layers and demonstrate specific biosensing of one of the endocrine system disruptors—the female hormone estrone—using the DBSPRI sensor.

Endocrine disrupting compounds, such as the hormone estrone, are especially prevalent in surface and waste-waters [26] in nano-molar concentrations, and therefore, there is a need for a sensitive analytical device for their monitoring. Hormones are chemical messengers that orchestrate many of the body’s internal functions—including cell growth, development and division—and how organs behave. They also handle communication between organs. These biologically active substances are secreted into the blood system via ductless glands of the endocrine system. They are active at very low concentrations (ng/mL to pg/mL, *i.e*., ppb or ppt) and bind specifically to target receptor sites on cell surfaces or within the cell nucleus. An endocrine disruptor (ED), such as estrone, is a synthetic compound that imitates a natural hormone when absorbed by the body. It can alter the normal hormone levels, activating excessive action or completely obstructing a natural response. In order to determine the level of the estrogenic disruptors in the environment, such as water, drink and food, as well as in human urine and serum, a sensitive and rapid method is needed. Although high-performance liquid chromatography (HPLC) [27] and gas chromatography-mass spectroscopy (GC-MS) [28,29] can be used for the analysis of estrone, several time-consuming sample pretreatment steps are required. Therefore, these methods are not suited for rapid analyses and also require a large volume of sample. When dealing with serum or urine, as the media of measurement, it would be very difficult to measure from the limited quantity available. To overcome these limitations, an enzyme-linked immunoassay (ELISA) [30] has been widely used for determination of estrogenic disruptors, due to its high selectivity and high sensitivity. However, ELISA requires more than one hour to complete a single measurement. Moreover, additional stages are needed, such as labeling. 

In this paper, we report on the immobilization study of antibodies on thin silver surfaces and detecting them using a DBSPRI sensor. We checked the quality of the chemistry process steps with the built-in lab refractometer based on excitation of surface plasmons (SPs). Molecular interactions on the silver nanolayers between probe and target shifted the SPR signal and, therefore, indicated the presence of the substances. At the last stage, we immobilized luminescence horseradish peroxidase (HRP) antibody and measured the luminescence signal with luminol and H_2_O_2_. We found that, incubation of silver thin films for 24 h while the concentration of 11-MUA is 10 mM shows smooth undamaged layers without aggregate formation. Once the protocol was developed and optimized, we immobilized rabbit anti-estrone polyclonal IgG on smooth thin silver layers. The specificity of the antibody we have used in this study is very high (7030-0604 (AbD Serotec Co.)); therefore, we can assume that nothing else rather than estrone was conjugated to the antibody. Experimental results from the Kretschmann-Raether set-up show that the binding of rabbit anti-estrone polyclonal IgG to 11-MUA caused the SPR angle to increase by 2.87°. Further addition of estrone to rabbit anti-estrone polyclonal IgG caused the SPR angle to increase by another 0.95°. The change of the SPR angle indicates the presence of the conjugated material and, therefore, the success of the immobilization. As the goal of the research, after immobilization, specific sensing of the endocrine disruptor—female synthetic hormone estrone—was demonstrated using a DBSPRI sensor. 

## 2. Experimental Section

### 2.1. Materials

**Chemicals:** All reagents were used as received without further purification. 11-Mercapto-undecanoic acid (11-MUA) 95%, 45056-1 Sigma Aldrich; dimethyl sulfoxide (DMSO) (Merck, Germany); ethanol 97%; N,N*'*-dicyclohexylcarbodiimide (DCC); EDC (1-ethyl-3-(3-dimethylaminopropyl)-carbodiimide) GR1-SI-E, 1769-0005 (Sigma-Aldrich Co., Israel); luminescence HRP antibody and luminol (Sigma-Aldrich Co., Israel); N-hydroxysuccinimide (NHS) GR1-FL-56480-0100 (Sigma-Aldrich Co., Israel); Phosphate buffered saline (PBS) pH 7.4; estrone E9750 (Sigma-Aldrich Co., Israel); rabbit anti-estrone polyclonal IgG 7030-0604 (AbD Serotec Co.); silicon adhesive isolators 3560-25EA (Sigma-Aldrich Co., Israel). 

**Evaporation tools:** Silver shot 1 to 3 mm, 99.9999% (00906Dz Sigma-Aldrich Co., Israel). Tungsten boat (Kurt J. Lesker Co.).

**Optical parts:** EO Laser Diode 6 mW 637 nm (59-088 Edmund Optics); BK7 right angle uncoated prism (FRP1309, Foctek); 1/4" CCD FireWire board level monochrome camera (NT58-223 Edmunds Optics); 25 mm focus length finite conjugate micro video lens, F2.5 (NT58-207 Edmunds Optics).

### 2.2. Preparation of Silver Samples

A thin smooth film of silver on BK7 glass substrates was grown. Using evaporative deposition, the silver and substrates were placed inside a vacuum chamber pumped down to 10^−6^ Torr. The resulting vapor then condensed on BK7 glasses inside the vacuum chamber. The shutter controlled the growth of the samples and shielded them from the initial burst of ‘crud’ that comes off of the silver source when it first melts. The film thickness is controlled with a crystal thickness monitor (CTM). Deposition rate is 2–5 Å/s.

### 2.3. Experimental Set-Up

#### 2.3.1. Refractometer Based on SPR for Testing of Immobilization Quality

**Figure 1:**
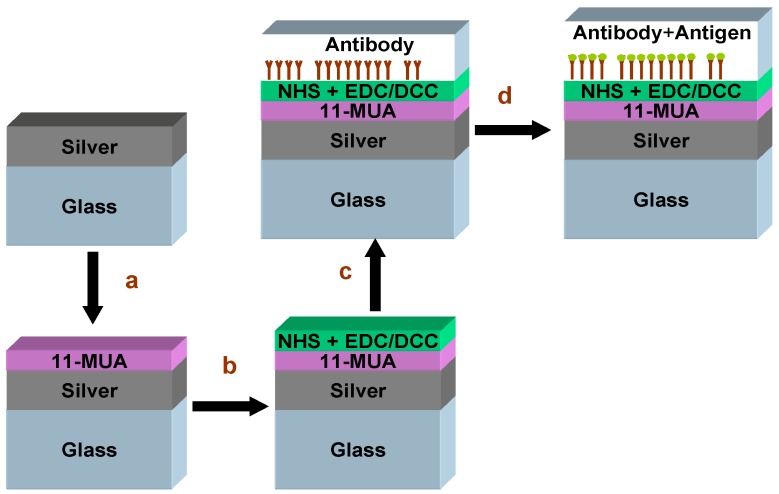
Schematic of the immobilization steps: (**a**) incubation in 11-MUA, (**b**) adding N-hydroxysuccinimide (NHS) + 1-ethyl-3-(3-dimethylaminopropyl)-carbodiimide (EDC) (or N,N*'*-dicyclohexylcarbodiimide (DCC)), (**c**) immobilization of antibody and (**d**) attachment of antigen.

We analyzed the refractive index (RI) changes at the end of each step of the immobilization protocol. The steps of the immobilization procedure are schematically shown in Figure 1. They included: (1) samples of smooth silver layers after deposition on glass, (2) incubation in 11-MUA of different concentrations and immersion times, (3) adding the NHS + EDC or NHS + DCC to make the silver surfaces reactive, (4) immobilization of the antibody and (5) attachment of the antigen. 

**Figure 2:**
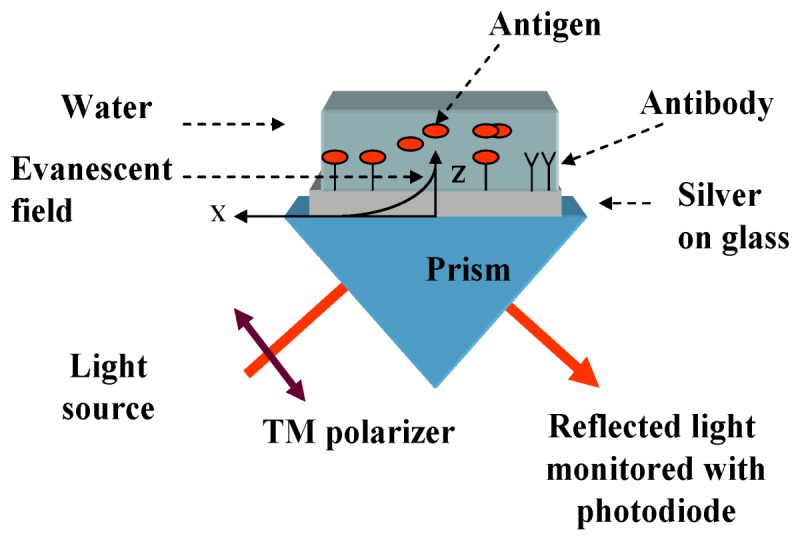
Kretschmann-Raether optical arrangement for examination of refractive index (RI) changes.

The refractometer used in our experiments was based on the Kretschmann-Raether (KR) configuration [31] for excitation of surface plasmons (SP). The set-up, which is shown in Figure 2, was constructed in our lab and contained the following components: a parallel-polarized monochromatic light source of 637 nm, a right angle prism and a silver thin film deposited on BK7 or SF11 glass and a photodetector. The silver film was placed at the interface of the prism and deionized (DI) water. A testing bath was attached to the silver film and was filled with DI water. When a light beam propagates in the prism and encounters the interface of the silver nanolayer, which was modified due to the immobilization, total internal reflection (TIR) takes place, and the evanescent wave forms, as long as the incident angle is greater than the critical angle *θ*_c_, where sin(*θ*_c_) = *n_analyte_*/*n_prism_*.

When the prism is fixed, the incidence angles are mechanically scanned, by changing the position of the light source and detector. Usually, the intensity of the reflected light does not change with the angle under the conditions of the TIR. However, at the particular angle greater than *θ*_c_, the evanescent wave excites the surface plasmons on the silver thin film surface in what is called the surface plasmon resonance (SPR). At this angle, which is called the SPR angle, *θ*_SPR_, the intensity of the reflected light decreases sharply. The angle required for resonance, *θ*_SPR_, is related to the RI of the analyte, *n_analyte_*, at a fixed RI of the prism, *n_prism_*, and silver, *n_silver_*:

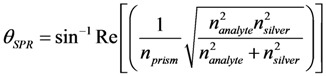
(1)


Adsorption on the silver surface (Figure 1) changes the RI of the analyte media near the metal, and therefore, *θ*_SPR_ changes accordingly. Therefore, measurements of the *θ*_SPR_ variations can be used to investigate the adsorption activities during the immobilization procedure steps. In addition to the SPR measurements, the last step of the immobilization process was measured using a luminometer, while immobilizing the luminescence HRP antibody to the silver surface, as is detailed in Section 2.4.1. 

#### 2.3.2. Diverged Beam SPR Imaging Set-Up

**Figure 3:**
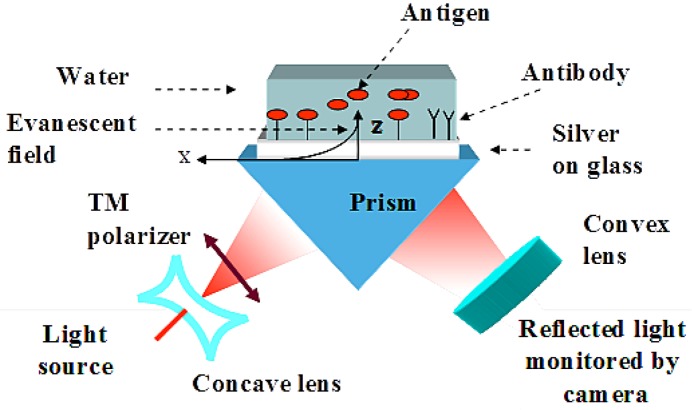
Prism coupling sensor and the diverged beam surface plasmon resonance (SPR) imaging (DBSPRI) arrangement scheme for improved angular interrogation without mechanical scanning.

Quasi-monochromatic laser light of wavelength 637 nm from the focusable laser diode was coupled to the plasmon modes at the metal-analyte interface with a variety of incident angels using a diverging incident light beam, which is in the *z*-*x* plan in the KR configuration by using a right angle BK7 or SF11 prism, as is shown in Figure 3 and explained thoroughly in our previous publications (see Karabchevsky *et al*.) [24,32]. The evanescent wave is generated at a single incidence-angle within the angular range of the diverged transverse-magnetic (TM) polarized beam. Reflected light is captured by the 659 × 494 pixels CMOS camera. When the momentum of the photons matches the momentum of the metal electrons (Equation (1) is satisfied), the dark line appears on the bright background of the captured images, which corresponds to the excitation of the surface plasmon.

### 2.4. Immobilization Procedure Based on Thiol Self Assembly Monolayer

#### 2.4.1. Protocol Based on Ethanol as a Solvent

11-mercaptoundecanoic acid (11-MUA) was dissolved in Ethanol in two different concentrations: 1 mM and 10 mM. A high concentration of MUA results in the formation of multilayers. Silver slides were immersed in different 11-MUA solutions for various immersion times: 4 h, 12 h, 24 h and 48 h, as is summarized in Table 2. Then, they were washed with ethanol and dried at room temperature. Twenty five millimolar of NHS and 100 mM of EDC were dissolved in Ethanol. The silver slides were immersed then in NHS/EDC solution for 30 min. After the immersion, the samples were washed with ethanol and dried at room temperature. For the immobilization assay, we used luminescence HRP antibody, which was immobilized on the silver thin films. HRP antibody (1:100 dilutions) was dipped on silver slides and was left for 1 h. Then, the slides were rinsed with PBS for 15 min and were left to dry in still air. Before the luminescence assay, H_2_O_2_ and luminol were dropped on the slides (50 μL: 50 μL). After immersion, samples were washed with ethanol. 

**Figure 4:**
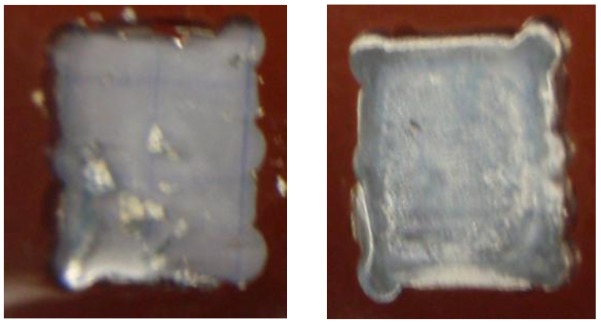
Samples after immobilization of luminescent horseradish peroxidase (HRP) antibody. The samples were immersed in 1 mM (**left**) and 10 mM (**right**) of 11-MUA for 24 h, and NHS with EDC was added prior the immobilization.

Table 2 implies that luminescence and SPR signals were achieved after immobilization of luminescent HRP antibody, while prior to the immobilization, the samples were incubated in the 10 mM 11-MUA for the period of 12 h, 24 h and 48 h. Therefore, the immobilization succeeded, while in the case of the incubation in 1 mM 11-MUA, the silver experienced major damage, as is documented by the camera and shown in Figure 4(left) together with the sample that was incubated for 12 h. However, there were some damaged areas on the surfaces of the silver that were immersed in 10 mM 11-MUA. In addition, after immersion and washing the samples with ethanol, a layer of aggregates was observed on top of the silver, as is demonstrated in Figure 4(right). This occurred because the Ethanol contains OH group which is known to form a stable bond with silver. Moreover, the affinity of the OH group to the silver is higher than those of thiol, therefore OH binds to Ag and forms visible aggregates. The luminescence and SPR signals were measured on the most smooth and undamaged areas of the samples.

**Table 2:** Summary of the scenario results: incubation times *versus* concentrations. Signs “v” and “-” indicate presence and absence of the signal respectively.

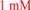 of 11-MUA		
Test	SPR signal in air	SPR signal in DI
Incubation time (h)		
4	v	silver was destroyed
12	v	-
24	v	-
48	v	-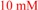 of 11-MUA		
Test	air in signal SPR	SPR signal in DI
Incubation time (h)		
4	v	v
12	v	v
24	v	v
48	v	v
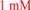 of 11-MUA + (NHS and EDC)		
Test	air in signal SPR	SPR signal in DI
Incubation time (h)		
4	silver was destroyed	-
12	silver was damaged partially	-
24	silver was damaged partially	-
48	silver was damaged partially	-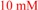 of 11-MUA + (NHS and EDC)		
Test	air in signal SPR	SPR signal in DI
Incubation time (h)		
4	silver was damaged partially	-
12	v	v
24	v	v
48	v	v
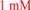 of 11-MUA + (NHS and EDC) + HRP			
Test	Luminiscence signal	air in signal SPR	SPR signal in DI
Incubation time (h)			
4	silver was destroyed	-	-
12	silver was destroyed	-	-
24	silver was destroyed	-	-
48	silver was destroyed	-	-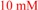 of 11-MUA + (NHS and EDC) + HRP			
Test	Luminiscence signal	air in signal SPR	SPR signal in DI
Incubation time (h)			
4	silver was damaged partially		
12	v	v	v
24	v	v	v
48	v	v	v

#### 2.4.2. Optimization of the Protocol with DCC and DMSO as a Solvent

Unlike ethanol, DMSO is an organic solvent without any functional group that can bind to the silver. In addition, DMSO is a suitable solvent for 11-MUA. In order to prevent the contact of oxygen with silver, which causes it to oxidize and deteriorates its plasmonic properties, we kept an inert atmosphere (without oxygen) using a stream of nitrogen, which is an inert gas.

11-MUA was dissolved in 10 mM of DMSO. After that, silver slides were immersed in the thiol solution under nitrogen atmosphere for 24 h. After the immersion period, the slides were washed with DMSO. Point-zero five molar NHS and 0.1 M EDC were dissolved in DMSO solution. The silver slides were immersed in EDC/NHS solution for 30 min and then washed with PBS. Rabbit anti-estrone polyclonal IgG antibody were dipped on silver slides (1:100 dilutions), kept for one hour and then washed with PBS. Since estrone is immiscible in water, we dissolved it in 1:5 by volume DMSO/DI in different concentrations. After that, silver slides were immersed in estrone solution overnight and then washed with PBS.

## 3. Results and Discussion

### 3.1. Specific Sensing of Estrone with DBSPRI Sensor

**Figure 5:**
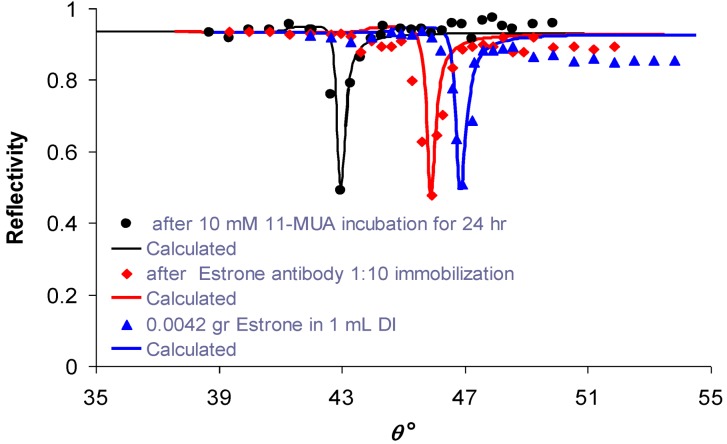
Experimental reflectivity *vs*. internal angle, *θ*, using the set-up depicted in Figure 2: circles—after incubation of the sample in 10 mM of 11-MUA for 24 h; diamonds—after immobilization of rabbit anti-estrone polyclonal IgG 1:10; triangles—after adding 0.0042 gr estrone in 1 mL DI. Calculated approximations for each signal are shown as smooth curves.

The silver surface was modified with 10 mM 11-mercaptoundecanoic acid (11-MUA) dissolved in DMSO. DCC and NHS were added to make the surface reactive, as was described in Section 2. Rabbit anti-estrone polyclonal IgG was then conjugated, making an ester bond with 11-MUA. As shown in Figure 5, the binding of rabbit anti-estrone polyclonal IgG to 11-MUA caused the SPR angle to increase by 2.87°. Further addition of estrone to rabbit anti-estrone polyclonal IgG caused the SPR angle to increase by another 0.95°. The change of the SPR angle indicates the presence of the conjugated material.

**Figure 6:**
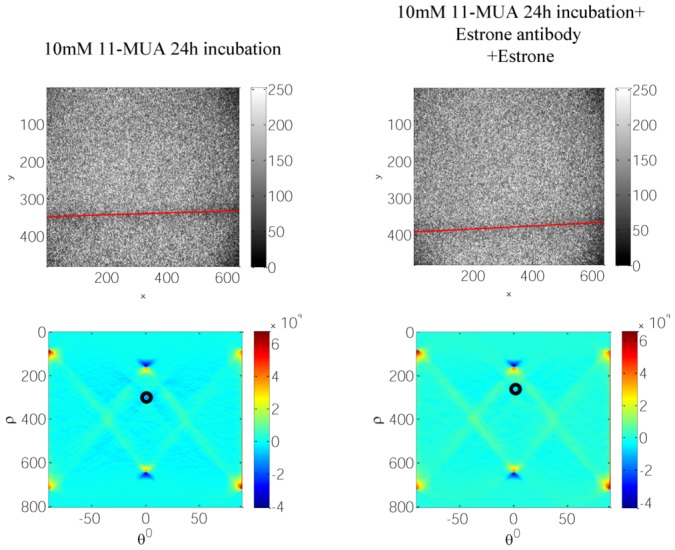
Reflected images from DBSPRI sensor, top: 68 nm Ag on BK7 glass after incubation in (**a**) 10 mM 11-MUA for 24 h and (**b**) after incubation in 10 mM 11-MUA for 24 h immobilized rabbit anti-estrone polyclonal IgG, while adding NHS + DCC prior to the immobilization; bottom: analyzed images in Radon space.

First, we validated the rabbit anti-estrone polyclonal IgG immobilization and attachment of the antigen—estrone—to it (Figure 5) with standard KR configuration, which is schematically shown in Figure 2. As is expected, the SPR angle increases as more molecules attached to the silver surface. After that, we demonstrated specific sensing of estrone with a DBSPRI sensor and analyzed the signals with a Radon transform-based algorithm, which was detailed in our previous work [24]. Figure 6 shows reflected signals from the DBSPRI sensor (depicted in Figure 3), while rabbit anti-estrone polyclonal IgG was immobilized on the silver surface. On top of Figure 6 is shown captured image, while 68 nm Ag on BK7 glass was incubated in (a) 10 mM 11-MUA for 24 h and (b) rabbit anti-estrone polyclonal IgG was immobilized, while adding NHS+DCC prior to the immobilization; down: analyzed images in Radon space.

**Figure 7:**
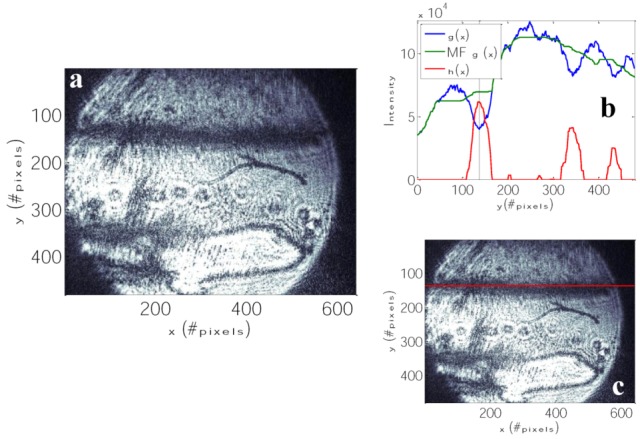
(**a**) Reflected images from DBSPRI sensor of 11-MUA + (NHS and DCC) after immobilization of estrone antibody and attached to it the estrone and after washing with PBS; (**b**) detection steps using the DBSPRI extraction algorithm [32]: blue—*g*(*x*), which is integrated original image; green—median filtered integrated image’ and red—median filtered image after background suppression. SPR line location is pointed by vertical lines; (**c**) reflected light, which was captured by the camera using the optical arrangement described in Figure 3, with extracted locations of the SPR signal, indicated by a solid line along the image.

Figure 7(a) shows the reflected image from the DBSPRI sensor described in Figure 3. Estrone antibody was immobilized and the antigen—estrone—was attached to it. Prior to the immobilization, the sample was incubated in 11-MUA for 24 h and NHS with DCC, which were added to make the surface reactive. Figure 7(b) shows detection steps using a multichannel extraction algorithm [32]: blue—*g*(*x*), which is integrated original image; green—median filtered integrated image; and red—median filtered image after background suppression. SPR line location is pointed by vertical lines. Figure 7(c) shows the extracted SPR line along the image. 

**Figure 8:**
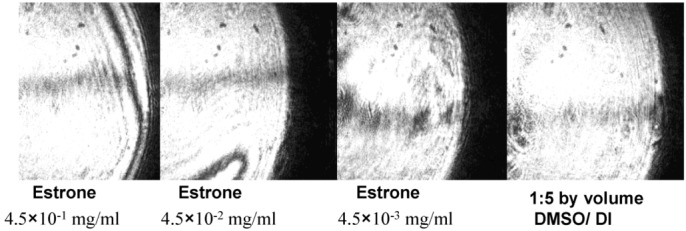
Reflected Images from the DBSPRI sensor with different concentrations of estrone 1:5 by volume dimethyl sulfoxide (DMSO)/deionized (DI) water used as a reference.

Figure 8 shows reflected images from the DBSPRI sensor after the immobilization procedure reported here, with different concentrations of estrone. PBS buffer was used as a reference.

## 4. Conclusions

Immobilization of antibodies on thin silver surfaces was investigated and optimized. The quality of the procedure steps were analyzed with the measurements of the changes in RI by means of the SPR angle changes. Molecular interactions at the surfaces between probe and target at the surface nanolayer shifts the SPR signal, thus indicating the presence of the substance. The last step of the immobilization procedure was tested with a luminometer using HRP antibody and luminol. We found that incubation of silver thin films for 24 h while the concentration of 11-MUA is 10 mM shows smooth undamaged layers without aggregate formation. Unlike ethanol, which is widely used, DMSO is also a suitable solvent for MUA and has no functional group that can react with the silver. In order to prevent the contact of oxygen with silver, which causes oxidation and deteriorates its plasmonic properties, we insisted on an inert atmosphere (without oxygen) using a stream of the nitrogen, which is an inert gas. 

Rabbit anti-estrone polyclonal IgG was successfully immobilized on smooth thin silver layers. Experimental results from the Kretschmann-Raether set-up show that the binding of rabbit anti-estrone polyclonal IgG to 11-MUA caused the SPR angle to shift. Further addition of estrone to rabbit anti-estrone polyclonal IgG caused the SPR angle to increase by another 0.95°. The change of the SPR angle indicates the presence of the conjugated material and, therefore, the success of the immobilization. As the goal of the research, after immobilization, specific sensing of the endocrine disruptor—female synthetic hormone estrone—was demonstrated using a DBSPRI sensor. 

Briefly, to conclude, in this work, we reported on: (i) the investigation and improvement of the immobilization protocol for attachment of antibodies on silver thin films, while the thickness of the silver layer is less than 70 nm, and (ii) the demonstration of the specific sensing of the endocrine disruptor—female synthetic hormone estrone—immobilized on silver surfaces using a DBSPRI sensor.
